# Internet-Delivered Tobacco Treatment for People Using Cannabis: A Randomized Trial in Two Australian Cannabis Clinics

**DOI:** 10.2196/14344

**Published:** 2020-12-07

**Authors:** Josephine Hindson, Tanya Hanstock, Adrian Dunlop, Frances Kay-Lambkin

**Affiliations:** 1 Hunter New England Health District Newcastle Australia; 2 University of Newcastle Callaghan Australia

**Keywords:** tobacco, cannabis, help-seeking behavior, internet-based intervention

## Abstract

**Background:**

Tobacco use is disproportionately higher in people who smoke cannabis than in the general population, increasing the severity of dependence for cannabis use, decreasing the likelihood of successful quit attempts for both cannabis and tobacco, and increasing the risk of relapse for both substances. Opportunities to address tobacco use in people using cannabis are being missed.

**Objective:**

This study aims to investigate the feasibility of engaging tobacco smokers who were accessing treatment for cannabis, with a tobacco-focused *i*nternet-based *He*althy *L*ifestyle *P*rogram (iHeLP; 4 modules). It was hypothesized that iHeLP completion would be associated with decreases in tobacco use and improved quality of life (QoL) and psychological health. It was also hypothesized that iHeLP completion would be higher in those who additionally received telephone support. Given that iHeLP aimed to improve healthy lifestyle behaviors, it was also hypothesized that there would be reductions in cannabis use.

**Methods:**

A total of 13 smokers seeking treatment for cannabis use were randomly allocated to iHeLP alone or iHeLP plus telephone support. Participants were engaged in iHeLP over 8 weeks and completed a 12-week follow-up assessment.

**Results:**

Results from 10 participants who completed the follow-up indicated that the acceptability of iHeLP was high-very high in terms of general satisfaction, appropriateness of services, effectiveness, and met need. Additional telephone support increased modal module completion rates for iHeLP from 0 to 2 but did not provide any other significant advantages over iHeLP alone in terms of cannabis use, tobacco use, QoL, or psychological health. Participants in the iHeLP-alone condition (n=4) reported a mean reduction of 5.5 (SD 9.00) tobacco cigarettes per day between baseline and follow-up, with a concomitant mean reduction in expired carbon monoxide (CO) of 5.5 parts per million (ppm, SD 6.91). The iHeLP plus telephone support group (n=6) reported a mean reduction of 1.13 (SD 4.88) tobacco cigarettes per day and a mean reduction of 9.337 ppm of expired CO (SD 5.65). A urinalysis indicated that abstinence from cannabis was achieved by 2 participants in the iHeLP-alone group and three participants in the iHeLP plus telephone support group. Between baseline and follow-up assessments, iHeLP-alone participants reported a mean reduction in days of use of cannabis in the prior month of 6.17 days (SD 13.30). The average reduction in the number of days of cannabis use for the iHeLP plus telephone support group was also 6.17 days (SD 13.59).

**Conclusions:**

Despite the small sample size, this study provides preliminary support for the use of internet-delivered, tobacco-focused interventions in tobacco smokers seeking treatment for cannabis use.

## Introduction

Cannabis is the most widely used illicit drug in the world [1], with up to 227 million people worldwide reporting the use of cannabis [2]. Australia has one of the highest rates of cannabis use in the world, with 10% of Australians older than 14 years reporting the use of cannabis in the previous 12 months [3].

Tobacco is another commonly used substance worldwide. It is estimated that in 2015, 21% of the adult population smoked tobacco in that year [4]. In Australia, 12.8% of people aged older than 14 years smoke tobacco, a reduction from 15.1% in 2010 [3]. Despite this, a number of subgroups in the Australian population still report relatively high rates of tobacco use. One such subgroup is the substance-using treatment-seeking population, with concurrent tobacco use estimates ranging from 74% to 100% [5].

There are generally high rates of tobacco smoking in people who smoke cannabis, including both concurrent and simultaneous uses. It is estimated that 50% of adults with cannabis use disorders are currently smoking tobacco, increasing a number of risks. Rates of dependence on cannabis when smoking tobacco are higher than when smoking cannabis only [6-8]. Combined cannabis and tobacco use also increases the dependence on tobacco [9], decreases successful quit attempts for both cannabis and tobacco [10-12], and increases the chance of relapse for both substances [13]. Combined cannabis and tobacco use also increases the risk of respiratory issues compared with smoking cannabis alone [8,14].

Only a small percentage of cannabis users access treatment [15,16]. Barriers to accessing treatment include the perception that cannabis use is not problematic enough to warrant treatment; concerns that quitting may exacerbate mental health and sleep- or pain-related issues [17,18]; reluctance to engage in traditional alcohol/other drug treatment services; notion that there are no specific treatment services for cannabis [19]; and avoidance of the stigma associated with being a *drug user* [18]. In contrast, cannabis users perceive tobacco as toxic and addictive [20] and rank it more harmful than alcohol or cannabis [21]. The majority of cannabis users report a clear intent to stop tobacco use [20], providing an opportunity for engaging them in discussions about their current lifestyle.

A large body of evidence exists for effective interventions that reduce and cease tobacco use, including pharmacotherapy and psychosocial therapies. For example, in a review of tobacco treatment research, Prochaska and Benowitz [22] reported that psychotherapies, delivered in both individual and group counseling settings, demonstrated effectiveness for tobacco cessation compared with self-help treatment. The appeal of psychosocial treatment is that strategies can be applied to both cannabis use and tobacco use simultaneously, providing a promising approach to addressing the co-occurrence of these behaviors. For example, Hoffman et al [23] conducted a review of 106 representative meta-analyses of cognitive behavioral therapy (CBT) for numerous disorders, including substance dependence. They found that similar CBT strategies were effective in the treatment of cannabis and nicotine dependence.

In the only study of its kind to date, Lee et al [24] investigated concurrent cannabis and tobacco computer-delivered treatment. They compared 32 cannabis-dependent adults who received treatment focused on both cannabis and tobacco to 54 participants, from a previous study, who had received either therapist- (n=28) or computer-delivered (n=26) treatment focusing only on cannabis. The treatment involved CBT, contingency management, and nicotine replacement therapy (NRT). The results indicated that the majority of participants were interested in receiving a tobacco intervention and that concurrent treatment for tobacco use did not compromise cannabis use outcomes. The tobacco intervention did not yield a high rate of abstinence at the end of treatment; however, it did motivate more than half the smokers to at least attempt to quit using tobacco.

A number of reviews have assessed the efficacy of internet-based treatment for tobacco use. Rooke et al [25] conducted a meta-analysis of 34 studies of 10,632 participants, and found that computer-delivered interventions significantly reduced tobacco and alcohol use overall, and that the magnitude of this change was comparable to individual counseling provided for tobacco use. In a Cochrane review of tobacco cessation programs, Civljak et al [26] analyzed 28 trials with over 45,000 participants involving any internet intervention. The comparison groups could include both internet and noninternet interventions. The results revealed that internet interventions that were tailored to the individual and interactive were more likely to aid in smoking cessation at 6 months posttreatment than those that did not. The authors suggested that younger people and women may be specifically interested in internet treatment; however, this requires more research.

A large study of adult tobacco smokers in the United States compared an internet-only intervention, where access to the internet-based intervention was given for 6 months, with an internet plus telephone support intervention for which the internet intervention was accompanied by five phone calls. The internet plus telephone support group reported significantly better outcomes up to 12 months of follow-up, suggesting that the addition of telephone support helped participants quit at an earlier time point that in turn had related health benefits [27].

Our team has recently developed an *i*nternet-delivered *He*althy *L*ifestyle *P*rogram (iHeLP) that targets tobacco use, in addition to other lifestyle factors of physical activity and healthy eating. The internet is increasingly becoming an option to deliver psychological treatment, especially CBT and motivational interviewing (MI), which are psychosocial treatment strategies with the strongest evidence for reducing both cannabis and tobacco use [28]. This study aimed to assess the feasibility of using iHeLP among tobacco smokers seeking treatment for cannabis use disorder by engaging with Specialist Cannabis Clinics in New South Wales (NSW) Health, Australia. Another aim of this study was to examine the role of telephone support in encouraging the uptake of iHeLP and to determine whether the use of the iHeLP program is associated with reductions in both cannabis and tobacco use.

## Methods

### Ethics

This study was designed as a feasibility study. It was approved by the Hunter New England Research Ethics and Governance Unit (HNEHREC Ref No: 15/05/20/4.06).

### Participants

Participants included 13 adults (7/13, 54% men; 6/13, 46% women) recruited from 2 Cannabis Clinics, one based within the Hunter New England Local Health District (HNELHD) and the other in the Central Coast Local Health District in NSW, Australia. Participants were aged between 20 and 50 years (mean age 34.15, SD 8.48 years). The eligibility criteria were patients older than 18 years, who had access to the internet via a personal device (computer, laptop, or mobile phone), who smoked at least five tobacco cigarettes per day, and who were not experiencing active psychosis at the time of recruitment. Intention to quit tobacco or cannabis was not a requirement for study participation. Participants could be at any stage of treatment for their cannabis use disorder, as long as they were currently attending appointments at the Specialist Cannabis Clinics [29].

### Procedure

Treating clinicians at both services initially screened patients for eligibility for the study. Those who met the eligibility criteria and were interested in participating were contacted within a week by the researcher (JH) to schedule an initial appointment and answer any questions regarding the study.

After obtaining informed consent, participants completed a 1-hour baseline face-to-face assessment. They were then randomized into one of the following 2 conditions: access to iHeLP (an internet-based healthy lifestyle program) with no support or access to iHeLP with telephone support. These interventions are described later. Research clinicians were blinded to treatment allocation until the conclusion of the baseline assessment was obtained.

Participants were provided with individual log-in details following randomization and had access to the iHeLP program for a period of 8 weeks. The participants were asked to complete the tobacco section of the program at a minimum and could choose to complete as many other sections as relevant. The participants were given Aus $20 (US $13.75) gift card for participation in the baseline assessment. Access to iHeLP and phone-based services were provided free of charge.

A follow-up face-to-face assessment was conducted at 12 weeks postbaseline. An Aus $20 (US $13.75) gift card was also offered to the participants who attended the follow-up appointment.

#### Treatment

Participants were randomized to iHeLP alone versus iHeLP plus telephone support. iHeLP targets tobacco use (2 modules), diet quality (1 module), and physical activity (including sedentary behavior; 1 module). Internet sessions are based on MI techniques and CBT strategies, as recommended for maximizing compliance. Within iHeLP, participants were asked to at least complete the tobacco modules as a starting point and then work their way through the remaining modules (physical activity and diet quality) in a sequence of their own, choosing over the 8-week treatment period. Automated emails were programmed to prompt module completion after a period of nonactivity. Participants accessed the treatment package from their home computer (or a preferred internet access port). The iHeLP intervention was not tailored for people using cannabis who also smoke tobacco, rather it incorporates standard tobacco cessation strategies and approaches that have demonstrated benefits in the general population and in people with mental health problems [30]. Please see Multimedia Appendix 1 for screenshots of the iHeLP program.

#### Telephone Support

Telephone support was provided to half of the study participants and involved 8 weekly phone calls on a set day and time to support access to and completion of iHeLP. Each call was limited to a duration of 10 min and was carried out manually. The content of such a phone call included reviewing the previous week, reinforcing the importance of making healthy lifestyle decisions, increasing adherence to the iHeLP modules, and discussing tobacco cessation attempts, including the use of NRT to manage tobacco cravings and withdrawal.

### Measures

Demographic information was collected at the baseline assessment. This included age, gender, education level, indigenous status, living arrangements, employment status, and source of income. The frequency of internet access was also measured. In addition, the following measurements were taken.

#### Depression Anxiety Stress Scale 21

The Depression Anxiety Stress Scale 21 (DASS 21) [31] was used at baseline and follow-up to assess current symptoms of depression (eg, “I felt I had nothing to look forward to”), anxiety (eg, “I felt I was close to panic”), and stress (eg, “?”). The validity and reliability of DASS 21 is sound, and a Cronbach of .90 was reported in existing literature for the depression subscale, and a Cronbach of .82 was reported for the anxiety subscale [32].

#### The Fagerstrom Test for Nicotine Dependence

The Fagerstrom Test for Nicotine Dependence (FTND) [33] was used for both assessments. FTND is a six-item scale, with scores ranging from 0 to 10, with higher scores indicating greater dependence. The reliability of FTND is good, with an acceptable Cronbach of .72 and a test-retest correlation of 0.82 [34].

#### Eurohis-QoL 8 Item

The Eurohis-QoL 8-item index [35] is a shortened version of the World Health Organization Quality of Life Instrument–Abbreviated Version and was used in both assessments. It measures the psychological, physical, social, and environmental domains on a 5-point Likert scale (0-5), with a total score between 8 and 40. In previous research, this instrument was found to have an acceptable Cronbach of .78 [35].

#### Australian Treatment Outcome Profile

The Australian Treatment Outcome Profile (ATOP) [36] is a standardized self-report measure used within the HNELHD Drug and Alcohol Clinical Services in NSW. It measures drug and alcohol use in the prior 4 weeks in terms of days used (frequency) and the average amount used (quantity). Self-reported frequency and quantity of cannabis and tobacco use in the prior 4 weeks were measured for all participants at baseline and follow-up. It should be noted that for this study, a *cone* (ie, *standard unit*) was used to estimate the quantity of cannabis use smoked on a use occasion. Cannabis can be shaped into a *cone* and smoked using a pipe or *bong*. Using this method, typically, one *cone* of cannabis is used per bong/pipe. Cannabis can also be rolled into a cigarette shape (a *joint*) and smoked like tobacco. For this study, it was estimated that the equivalent of 3 cones of cannabis was used per *joint*. Participants were asked to estimate the number of *cones* of cannabis they used per use occasion across both *bongs*/*pipes* and *joints*. A *use occasion* was defined as a period of using cannabis from the time a person started smoking until the time they finished smoking. A person could report more than one *use occasion* in a day. ATOP also contains questions regarding days worked and studied in the previous 4 weeks. It has yes/no questions regarding housing situations, children in care, violence, and arrests in the past 4 weeks. It uses a 10-point Likert scale (0-10) to measure the psychological health, physical health, and overall quality of life (QoL). The scale has an acceptable concurrent validity and interrater reliability in the established literature [36].

#### Credibility/Expectancy Questionnaire

The Credibility/Expectancy Questionnaire [37] was administered at baseline only to determine the extent to which participants perceived internet-based treatments as a credible form of treatment for substance use and to ask them to estimate the expected outcomes associated with the completed treatment for substance use internet-based. Responses were rated on a 9-point Likert scale (eg, 1=not at all logical/confident/useful to 9=very logical/confident/useful), and estimated improvements were nominated from 0% to 100%. The psychometric properties have been previously reported, and it was found that the expectancy factor had a standardized alpha of between .79 and .90, the credibility factor had a Cronbach between .81 and .86, and the whole scale had a standardized alpha of between .84 and .85 [37].

#### Client Satisfaction Questionnaire

The Client Service Questionnaire (CSQ) [38] was administered at follow-up only. This is an eight-item measure that includes both numerical questions and the possibility of providing feedback on the treatment received. All response options are based on a four-point scale. The psychometric properties have been previously assessed [38] with high internal consistency (Cronbach =.93) and construct validity (ranging from 0.6 to 0.8) reported.

#### Expired Carbon Monoxide

The Bedfont piCO+Smokerlyzer (Bedfont Scientific) was used to assess expired carbon monoxide (CO) at baseline and follow-up assessments and is an objective measure of the current tobacco use status. Previous research has shown that expired CO is a reliable measure for validating self-reported tobacco use [39]. A reading of 10 parts per million (ppm) or higher is indicative of continued tobacco use.

#### Urinalysis

A urinalysis was used to confirm self-reported abstinence from illicit substance use at both assessments. DipScan measures amphetamines, benzodiazepines, cocaine metabolites, methamphetamines, opiates, and cannabis metabolites at cutoff levels defined by the Australian Standard AS/NZS 4380:2008.

#### iHeLP Website Analytics

The iHeLP program tracked the participant’s progress through each module to obtain an objective measure of the extent of engagement (ie, program completion) associated with the program.

### Statistical Analysis

The main focus of the statistical analysis was descriptive and was conducted using SPSS Statistics Software (version 22.0, IBM Corporation). To address engagement and completion of iHeLP, module completion rates were calculated for each condition (iHeLP vs iHeLP plus telephone support), based on the number of modules completed (not attempted or started=0).

To address changes in cannabis use (ATOP), tobacco (ATOP), QoL scores, DASS 21, and DASS 21 anxiety scores, paired sample *t* tests were used to analyze baseline to follow-up changes in each variable for the sample as a whole. Statistical tests for differential changes in these variables as a function of treatment allocation were not performed due to the small sample size. Instead, descriptive statistics were reported for each of these variables for baseline and follow-up completers according to treatment allocation.

## Results

### Demographics

A total of 13 participants were enrolled in the program over a period of 3 months and completed the baseline assessment. The mean age of the sample was 34.15 years (SD 8.484 years; range 20-50 years), and there were 7 men and 6 women. As indicated in Table 1, 3 (3/13, 23%) participants were identified as being Aboriginal or Torres Strait Islander. The majority of participants were unemployed, 2 had full-time jobs, and 2 had part-time jobs (Table 1). The mean number of days worked in the last 4 weeks was 6.15 (SD 8.69 days; range 0-20 days), and the average number of days studied in the previous 4 weeks was 2.00 (SD 5.65 days; range 0-20 days). At baseline, internet access ranged from several times a day, every day, and several times a week to once a month or less. See Table 1 for demographic details of the participants.

**Table 1:** Baseline participant characteristics (n=13).

Demographic characteristics		Values
Age (years), mean (SD)		34.15 (8.484)
Gender (male), n		7
Aboriginal or Torres Strait Islander, n		3
**Highest level of education, n**		
	Technical College (technical diploma/certificate)	9
	High school	2
	Did not complete high school	2
**Employment status, n**		
	Full-time work	2
	Part-time work	2
	Household duties	1
	Unemployed	8
**Source of income, n**		
	Wage/salary	3
	Own business	1
	Government benefits	7
	Family payments (government)	2
**Internet access, n**		
	Several times a day	6
	Every day	2
	Several times a week	4
	Once a month or less	1

**Figure 1:**
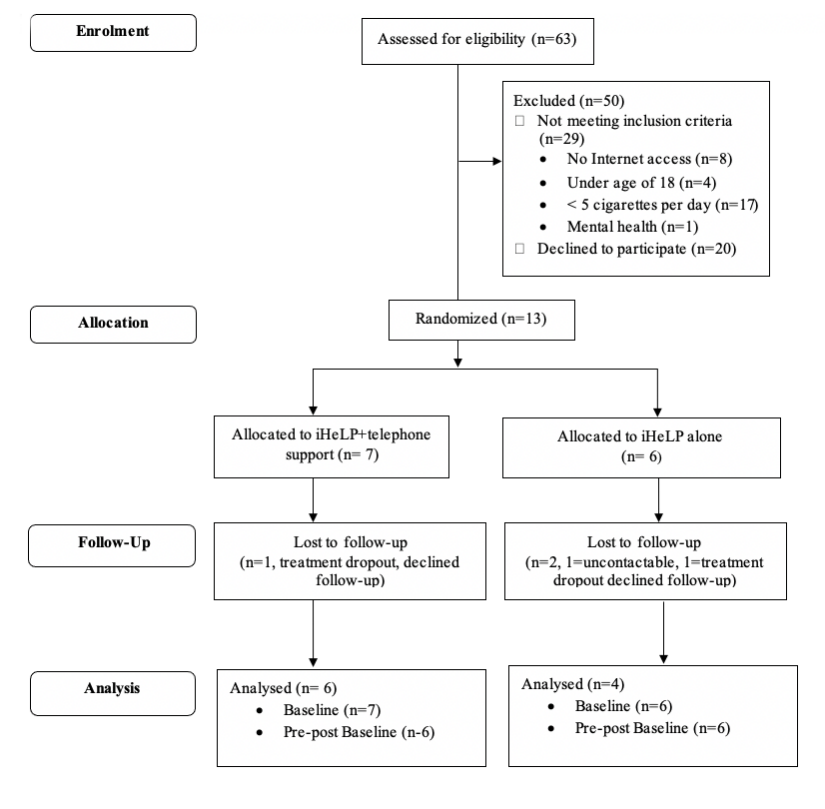
Flow of participants through the study.

Of the 13 people recruited to the study, 6 were randomized to iHeLP alone, while 7 were randomized to the iHeLP plus telephone support group. Of those who entered the study, 4 from the iHeLP alone and 6 from the iHeLP plus telephone support group completed the study. The recruitment and study enrollment details are shown in Figure 1.

### Substance Use

At baseline, the mean value for FTND was 5.15 (SD 2.30; range 0-9; medium dependence), the average expired CO was 25.00 (SD 12.65; range 4-53), and the average tobacco use for the past month was 16.77 (SD 7.88; range 5-30) cigarettes per day. The average number of days of cannabis use in the past month was 17.54 (SD 13.14 days), and the average amount of cannabis consumed in each use occasion was 11.08 (SD 11.94 cones). At baseline, the average depression score on DASS-21 was 12.92 (SD 10.60; *mild*), and the average DASS-21 anxiety score was 12.46 (SD 9.28; *moderate*).

### Participants’ Engagement and Acceptance of iHeLP

Participants in the iHeLP-alone condition attended a mean of 6.75 sessions (SD 5.25) at their respective cannabis clinics. Participants in the iHeLP plus telephone support condition attended a mean of 6.83 sessions (SD 3.37). The mean number of modules completed by the iHeLP program for the iHeLP-alone group was 1.17 modules (SD 1.33; range 0-3) and 1.85 modules (SD 1.46; range 0-4) for the iHeLP plus telephone support group. Participants in the iHeLP plus telephone support group received an average of 6.83 (SD 0.98) supportive phone calls over the 8-week treatment period, lasting for an average of 5.50 (SD 1.76) min each.

People in the iHeLP-alone condition reported a modal session completion of 0 (median 1.00; SD 1.17). In the iHeLP plus telephone support group, 2 modal modules were completed (median 2.00; mean 1.85, SD 1.12; Table 2).

**Table 2:** Frequency of participants completing each module of the iHeLP program as a function of study condition (iHeLP alone versus iHeLP enhanced).

	iHeLP alone	iHeLP enhanced
Module number completed		
0	3	2
1	0	0
2	2	3
3	1	1
4	0	1

Overall, the participants reported being satisfied with iHeLP. As can be seen in Table 3, all mean satisfaction scores on the CSQ were between 3 and 4 for both the iHeLP-alone and iHeLP plus telephone support conditions. This indicates that there was a high to very high satisfaction with the program. Average *general satisfaction* was rated by iHeLP-alone participants as 4.00 (*very satisfied*) and iHeLP plus telephone support as 3.83 (SD 0.41; *mostly satisfied to very satisfied*; n=6). Average *appropriateness of services* was rated by iHeLP-alone participants as 4.00 (*highly appropriate*) and as 3.83 for the iHeLP plus telephone support group (SD 0.41; *generally appropriate to highly appropriate;* n=6). Table 3 displays the satisfaction ratings across all domains measured according to the treatment allocation.

**Table 3:** Mean ratings on the Client Service Questionnaire provided by participants, as a function of study condition (iHeLP alone versus iHeLP enhanced)^a^

Program	Promptness	Focused on helping versus procedures	Quality of service	Service aligned with wants	Service met needs	Recommend service to a friend?	Satisfaction with help received	Services helped deal with problems more effectively	Overall	Would you return?	Service appropriate	Problems now?	Kept info private?
iHeLP alone	4.00	4.00	4.00	4.00	3.25	4.00	4.00	3.50	3.75	4.00	3.75	3.50	4.00
iHeLP enhanced	3.67	3.50	3.83	3.50	3.33	3.83	3.83	3.50	3.83	3.83	3.83	3.33	4.00

^a^response categories range from 1 to 4, with higher scores indicating higher satisfaction.

### Changes in Tobacco Use Over Time

Paired sample *t* tests for the overall sample (n=10) revealed that FTND scores decreased from 5.7 (SD 1.95) at baseline to 4.6 (SD 2.55) at follow-up. This difference was statistically significant (*t*_9_=2.283; *P*=.048).

**Figure 2:**
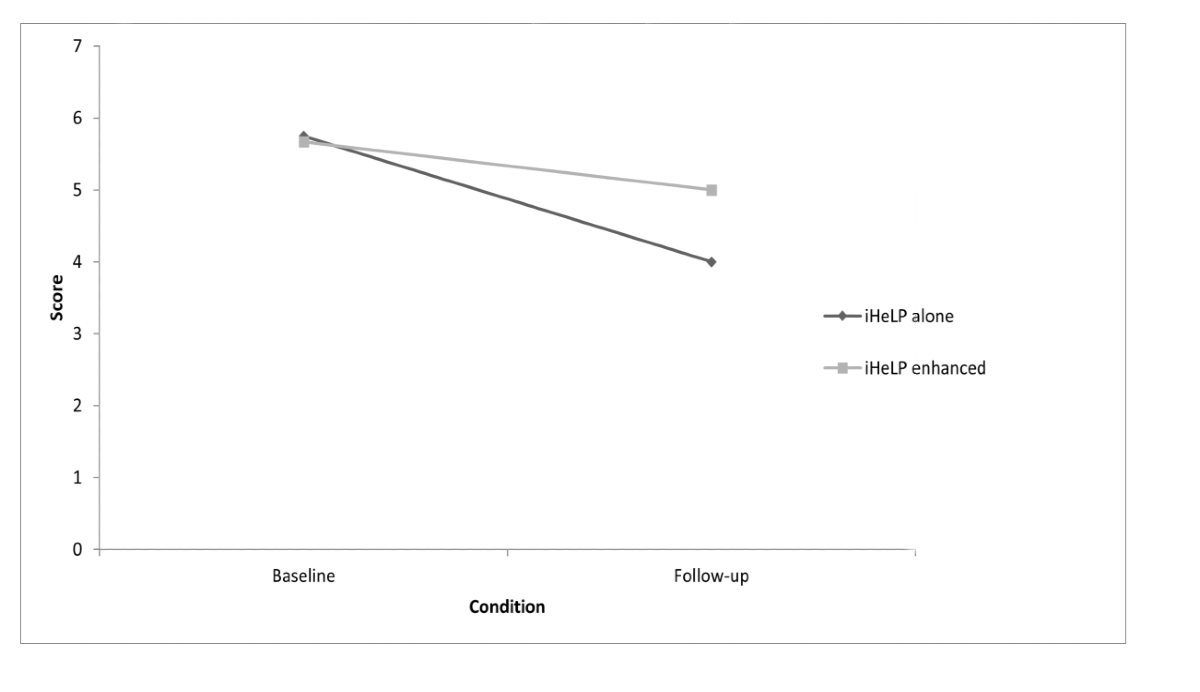
Changes in scores on the Fagerstrom test for nicotine dependence between baseline and follow-up assessment, according to treatment allocation.

The 4 participants in the iHeLP-alone group reported an FTND baseline mean of 5.750 (SD 1.03), and at follow-up, this had decreased to a mean of 4.00 (SD 1.32). The iHeLP plus telephone support group reported an FTND baseline mean of 5.67 (SD 0.84), which decreased to 5.00 (SD 1.08; Figure 2).

For the 10 participants completing follow-up assessments, the number of tobacco cigarettes smoked per day decreased from a mean of 18.4 (SD 7.53) at baseline to 15.5 (SD 6.40) at follow-up. Participants also reported a reduction in days of tobacco use in the month before assessment, decreasing from a mean at baseline of 28 (SD 0.00) days to 26.40 (SD 5.06) days at 12-week follow-up. Objective measures of expired CO supported these self-reported reductions in tobacco, with the mean expired CO for the 10 participants decreasing from 25.60 ppm (SD 14.546) at baseline to 17.80 ppm (SD 13.054) at follow-up.

The 4 iHeLP-alone participants who completed follow-up assessments reported a baseline mean expired CO reading of 22.50 ppm (SD 7.58), which reduced to 17.00 ppm at follow-up (SD 6.91). The iHeLP plus telephone support group reduced from 27.667 ppm expired CO (SD 6.19) at baseline to 18.33 ppm (SD 5.65) at follow-up.

**Figure 3:**
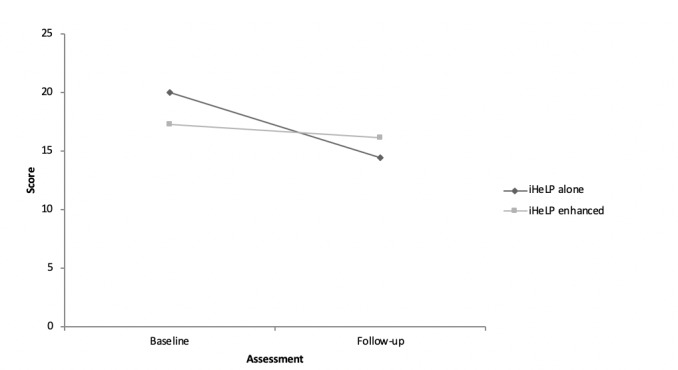
Changes in self-reported tobacco use (cigarettes per day) between baseline and follow-up assessments, according to treatment allocation.

At baseline, the 4 iHeLP-alone participants who completed both assessments reported a mean daily cigarette consumption in the prior month of 20.00 (SD 12.25). This reduced to a mean of 14.50 (SD 9.00) at follow-up assessment. The 6 iHeLP plus telephone support participants who completed both assessments reported a reduction in past month cigarettes per day from a mean of 17.33 (SD 2.94) at baseline to a mean of 16.17 (SD 4.88) at follow-up (Figure 3).

### Changes in Cannabis Use Over Time

The 10 participants providing follow-up data reported a median of 10.00 cannabis cones (mean 11.08, SD 11.94) per day at baseline in the month before the assessment, and this reduced to a median of 3 (mean 7.0, SD 11.56) per day at follow-up. Participants also reported a reduction in cannabis use days in the month before the assessment with a median at baseline of 28 days (mean 17.54, SD 13.14), which reduced to a median of 4 days (mean 10.90, SD 12.88). The urinalysis confirmed self-reported abstinence from cannabis use on all occasions. Of the 10 follow-up participants, 8 provided follow-up urine samples for analysis, of which 5 self-reported abstinence from cannabis, and the urinalysis was negative. Of the remaining patients, 3 self-reported continued use of cannabis, and all returned a positive urinalysis, indicating continued use. The 2 patients who declined to provide a urine sample self-reported continued use of cannabis at follow-up.

For the 4 participants in iHeLP alone who provided follow-up data, cannabis use in the prior month remained reasonably constant, with a baseline mean of 5 cones per day (mean 5.25, SD 6.58) and a mean of 5.25 cones per day (SD 6.08) at follow-up. Two participants in the iHeLP-alone group returned a negative urine sample, indicating abstinence from cannabis. The iHeLP plus telephone support group (n=6) reported reductions from a mean of 11 cones per day (mean 11.33, SD 6.58) at baseline to a mean of 8 cones per day (mean 8.18, SD 4.96) at follow-up. A negative urinalysis was returned for 3 of these participants, indicating abstinence from cannabis use.

The number of days cannabis was used in the iHeLP-alone group (n=4) reduced from a median of 16 days at baseline (mean 14.67, SD 13.125) to 3 days (mean 8.50, SD 13.30) at follow-up. The iHeLP plus telephone support group (n=6) reported a reduction in days of cannabis use from a median of 28 (mean 18.67, SD 14.46) days at baseline to a median of 10 (mean 12.50, SD 13.59) days at follow-up.

### Participant Quality of Life Over Time

The participants who provided follow-up data (n=10) reported a mean of 22.6 (SD 4.6) at baseline for their current QoL, and this significantly increased to 27.40 (SD 4.12) at follow-up. For the 4 participants in iHeLP alone, QoL ratings increased from 20.50 (SD 2.24) at baseline to 29.50 (SD 1.96) at follow-up. The iHeLP plus telephone support group also reported an increase in QoL scores from 24.00 (SD 1.83) at baseline to 26.00 (SD 1.6) at follow-up.

### DASS-21 Scores Over Time

The 10 participants providing follow-up data reported mean DASS 21 scores of 12.60 (median 12.00, SD 11.74) at baseline, and this decreased to 7.40 (median 8.00, SD 6.33) at follow-up. For the 4 iHeLP-alone participants, the mean baseline DASS-21 depression scores were 21.00 (median 20, SD 4.91) and 10.00 (median 11, SD 3.14) at follow-up. The iHeLP plus telephone support group reported a mean DASS-21 depression score of 7.00 (median 4, SD 4.01) at baseline, and this decreased to 5.67 (median 6, SD 2.56) at follow-up.

For DASS-21–anxiety, the 10 participants providing follow-up assessments reported a mean baseline score of 11.40 (median 10, SD 10.16), which decreased to 5.60 (median 5, SD 4.40) at follow-up (*t*_9_=1.99; *P*=.08). Within the iHeLP-alone group (n=4), the mean baseline DASS-21–anxiety scores were 18.00 (median 19, SD 4.47), which decreased to a mean of 5.50 (median 4, SD 2.33) at follow-up. The iHeLP plus telephone support group (n=6) reported at baseline a DASS-21–anxiety score of 7.00 (median 8, SD 3.65), which decreased at follow-up to 5.67 (median 6, SD 1.91).

## Discussion

### Principal Findings

This study is the first of its kind to explore the use of an internet lifestyle intervention in tobacco smokers seeking treatment for cannabis use disorder. It has shown that participants offered the program would engage with the program even if they are not seeking treatment for lifestyle factors such as tobacco use. It has also been shown to have benefits for tobacco use and QoL.

The primary aim of this study is to explore the feasibility of iHeLP among tobacco smokers who were accessing treatment for cannabis use. The secondary aim was to evaluate if additional telephone support provided by a psychologist increased the engagement and completion rates of iHeLP in this sample. The clinical outcomes of interest were cannabis and tobacco use as well as QoL and psychological health, hypothesizing that cannabis and tobacco use would decrease while QoL and psychological health would improve over the course of the study.

The main finding of this study was that iHeLP was highly acceptable as seen by the high levels of satisfaction reported by the participants. The study also found that the participants did access iHeLP, with half of the total number of participants completing at least one module, which comprised the tobacco-related component of the internet-based program. This is significant, given that the participants were accessing the cannabis treatment clinic for their cannabis use specifically and were not seeking treatment for their tobacco use. This provides some initial support for the value of offering cannabis users an opportunistic intervention for tobacco use when they present for treatment. Although half of the study participants reported use of the internet every day (and for some, several times a day), 5 out of 13 participants were less frequent users of the internet at the beginning of the study. Internet access at baseline was not associated with the uptake or use of the iHeLP program, demonstrating the potential of internet-delivered interventions even with infrequent users of this technology.

### Comparison With Prior Work

When participants had the addition of telephone support from a psychologist to iHeLP (iHeLP plus telephone support), there were no apparent increases in the rates of module completion. However, when looking at the modal number of sessions completed in each treatment group, those in the iHeLP plus telephone support group were most likely to complete two modules, with the mode for the iHeLP-alone group being 0. Thus, there is some suggestion here that therapist assistance (even as little as 5 min per week as provided in this study) did seem to coincide with the greater uptake with iHeLP. This is consistent with the previous research in internet-based interventions, which indicates that the addition of therapist support is associated with higher quit rates and more active involvement in the program [40,41].

Reductions in both tobacco dependence scores and the amount of tobacco smoked per day were reported by participants across both iHeLP conditions. In the total sample providing follow-up data (n=10), this reduction corresponded to dependence scores of *medium* at baseline to *low* dependence at 12-week follow-up, a clinically significant change in levels of tobacco dependence. Previous research has indicated that internet interventions can be as effective as counseling in producing a reduction in tobacco use, and those that are interactive and tailored to the individual produce better results [42,43]. This study adds to this existing research by demonstrating that internet interventions for tobacco use are feasible and can be considered as another treatment option for people accessing treatment for cannabis.

Cannabis use did decrease between baseline and follow-up, in terms of actual cannabis consumption and days of use. This is expected, given that participants were actively engaged in treatment for their cannabis use throughout the study, and had attended an average of 6 sessions with the specialist cannabis clinic. These cannabis reduction rates are consistent with other studies and highlight the difficulties clinicians and individuals face in encouraging changes in cannabis use [44]. These difficulties include retention in treatment and engagement [45]. Together, this body of evidence suggests that a continued and major effort is required to better understand the nature of cannabis use and how to encourage change in cannabis use in those seeking treatment.

QoL ratings improved for the sample providing follow-up data, indicating the potentially broader benefits of a lifestyle intervention in tobacco smokers seeking treatment for cannabis use disorders. Important clinical improvements were also reported by the participants in terms of their depression and anxiety over time. For DASS-21–depression, the iHeLP-alone group in particular were in the *severe* range at baseline, and this dropped to a *mild* rating at follow-up. This pattern was also observed in the iHeLP-alone group for DASS-21–anxiety, who decreased from a *severe* rating at baseline to a *mild* rating at follow-up. As there is a large difference between baseline scores for depression in the 2 groups in this study, future studies should consider using depression as a stratification variable or control for depression in the analysis.

### Limitations

This study had a number of methodological issues. The major limitation of this study was the small sample size; therefore, the results need to be interpreted with caution. Recruitment proved difficult for a number of reasons. A large percentage of clients were unable to access the internet, with 26.7% of eligible clients reporting no access. This is in line with previous concerns regarding the limitations of internet interventions for this particular patient population [46]. Although catering to the proportion of cannabis users without internet access is critically important, internet interventions can be accessed by a large proportion of cannabis users. It should also be noted that at this stage, the iHeLP program was not accessible on mobile devices and this may have prevented some potential and willing participants from engaging with the study. Of the eligible sample with internet access in this study, despite reporting significant social disadvantage, 82% (10/13) reported at least weekly use of the internet. This provides at least partial support for the further exploration of internet-based treatment programs in people seeking treatment for cannabis use. Over half (58.6%) of individuals screened for this study did not meet the criteria for tobacco use. This percentage was much higher than expected from previous research [47]. This may be indicative of the knowledge that participants in studies comparing the perceptions of cannabis and tobacco have found that tobacco is perceived as harmful and toxic, and accordingly, cannabis users have already modified their tobacco use [21,48].

It should be noted that the researcher of this study (JH) was also a treating clinician for a percentage of participants (30%) at one of the recruitment sites for the study. While steps were taken, in line with ethics approvals, to reduce the perceptions of the coercion, these results may be subject to bias and should be interpreted with caution.

Finally, this study was conducted with cannabis users who already sought treatment for their cannabis use. It is not known how an internet intervention for tobacco use might appeal to or engage in nontreatment seekers for cannabis use and similarly whether internet access exists for cannabis users not accessing treatment.

### Clinical Implications

Notwithstanding the significant limitations of this study, there is some initial support for the clinical value of integrating internet tobacco programs into substance use services, particularly in encouraging tobacco reduction in cannabis users. Future studies could investigate the efficacy of NRT in conjunction with internet programs to evaluate the efficacy of these treatment options in encouraging abstinence from tobacco in people using cannabis. It is also important to investigate the role of additional support alongside the internet-based treatment to increase adherence to and completion of similar internet-based programs for cannabis users.

As shown in previous studies, those accessing treatment for cannabis usually present with a more complex profile than those cannabis users who do not present for treatment [49]. Thus, this study should be replicated with a non–treatment-seeking sample of cannabis users who are concerned about their tobacco consumption. It is well documented that tobacco is perceived as more harmful than cannabis by cannabis smokers [20,50] and thus may be a more acceptable treatment option for cannabis users not accessing treatment. Early success with tobacco cessation, particularly via a broader *healthy lifestyle* approach, may act as a gateway to considering changes in other *lifestyle* areas (eg, cannabis use), thus encouraging treatment seeking.

### Conclusions

The results from this study support the acceptability of an internet-based iHeLP (lifestyle-focused) program for tobacco smokers seeking treatment for cannabis use disorders. The addition of telephone support by a psychologist did not produce any significant advantages, apart from a higher modal completion rate in those with additional therapist support. There was a statistically significant reduction in nicotine dependence over the study period, which has significant public health and physical health implications. The results of this study are encouraging regarding the acceptability of the iHeLP program on a subgroup of the population who are both difficult to engage and often excluded from research studies.

## Appendix

Multimedia Appendix 1 Screenshots of the iHeLP Program.

## Abbreviations

ATOP: Australian Treatment Outcome Profile
CBT: cognitive behavioral therapy
CO: carbon monoxide
CSQ: Client Service Questionnaire
DASS: Depression Anxiety Stress Scale
FTND: Fagerstrom Test for Nicotine Dependence
HNELHD: Hunter New England Local Health District
iHeLP: internet-delivered Healthy Lifestyles Program
MI: motivational interviewing
NRT: nicotine replacement therapy
NSW: New South Wales
QoL: quality of life
