# Supplementary material for: Internet-Delivered Tobacco Treatment for People Using Cannabis: A Randomized Trial in Two Australian Cannabis Clinics
Source: JMIR Form Res. 2020 Dec 7;4(12):e14344. doi: 10.2196/14344 (PMC7752536; doi:10.2196/14344)
Supplement: Multimedia Appendix 1 [file formative_v4i12e14344_app1.pdf]

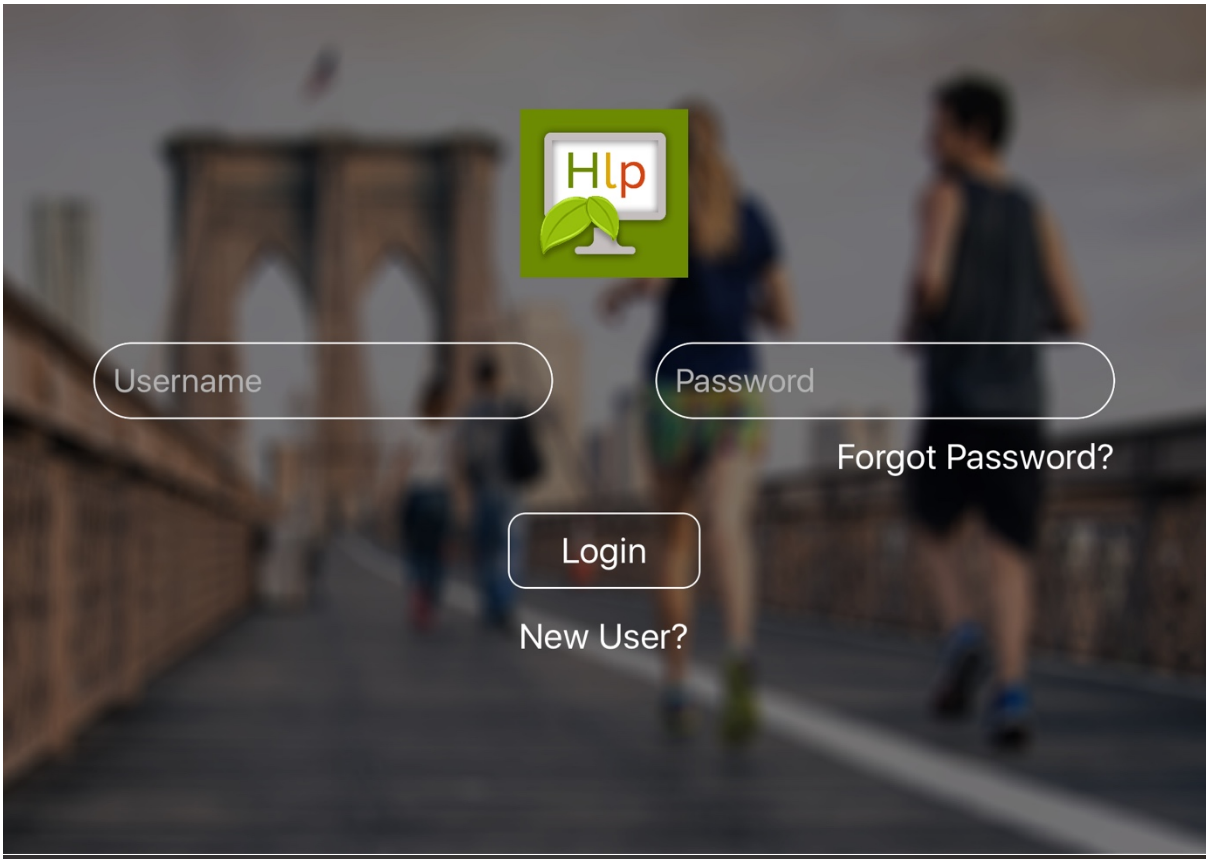
Hlp
Username
Password
Forgot Password?
Login
New User?

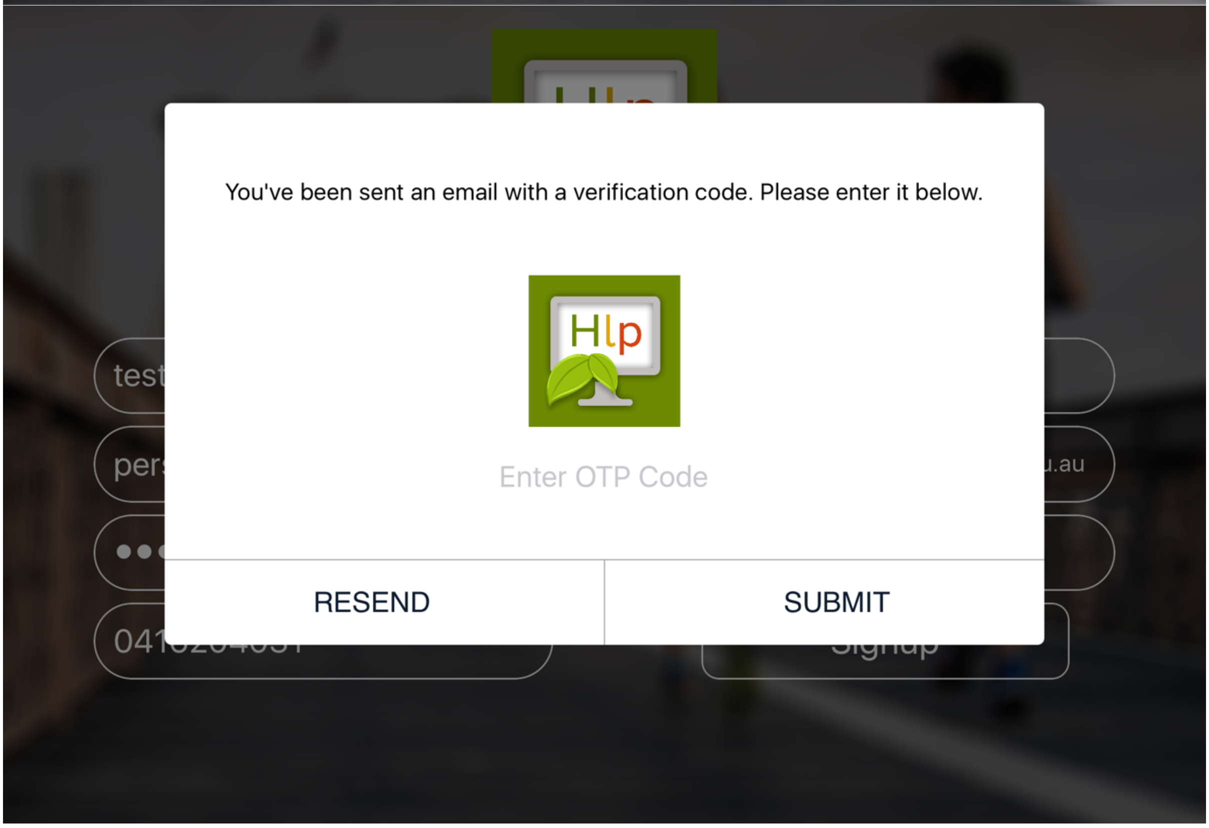
You've been sent an email with a verification code. Please enter it below.
Hlp
Enter OTP Code
RESEND
SUBMIT

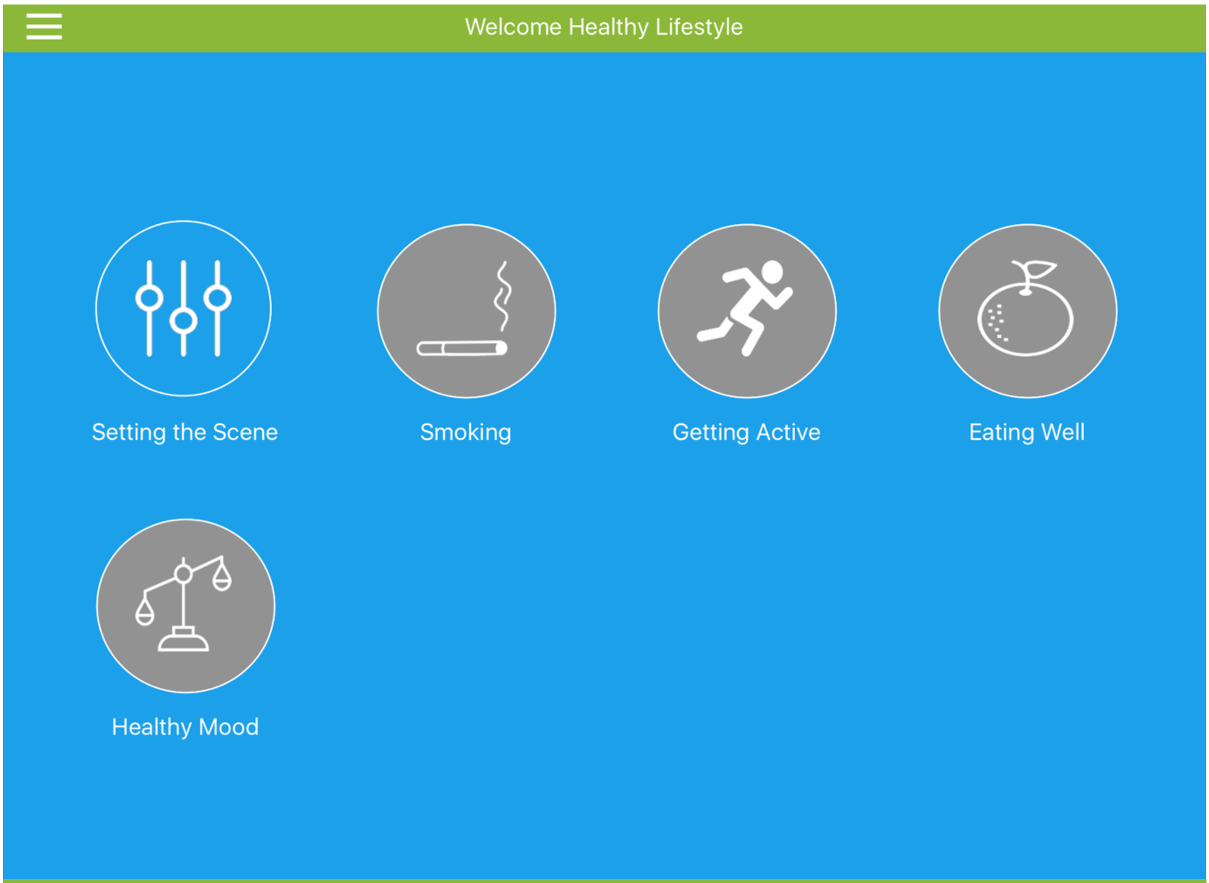
Welcome Healthy Lifestyle
Setting the Scene
Smoking
Getting Active
Eating Well
Healthy Mood

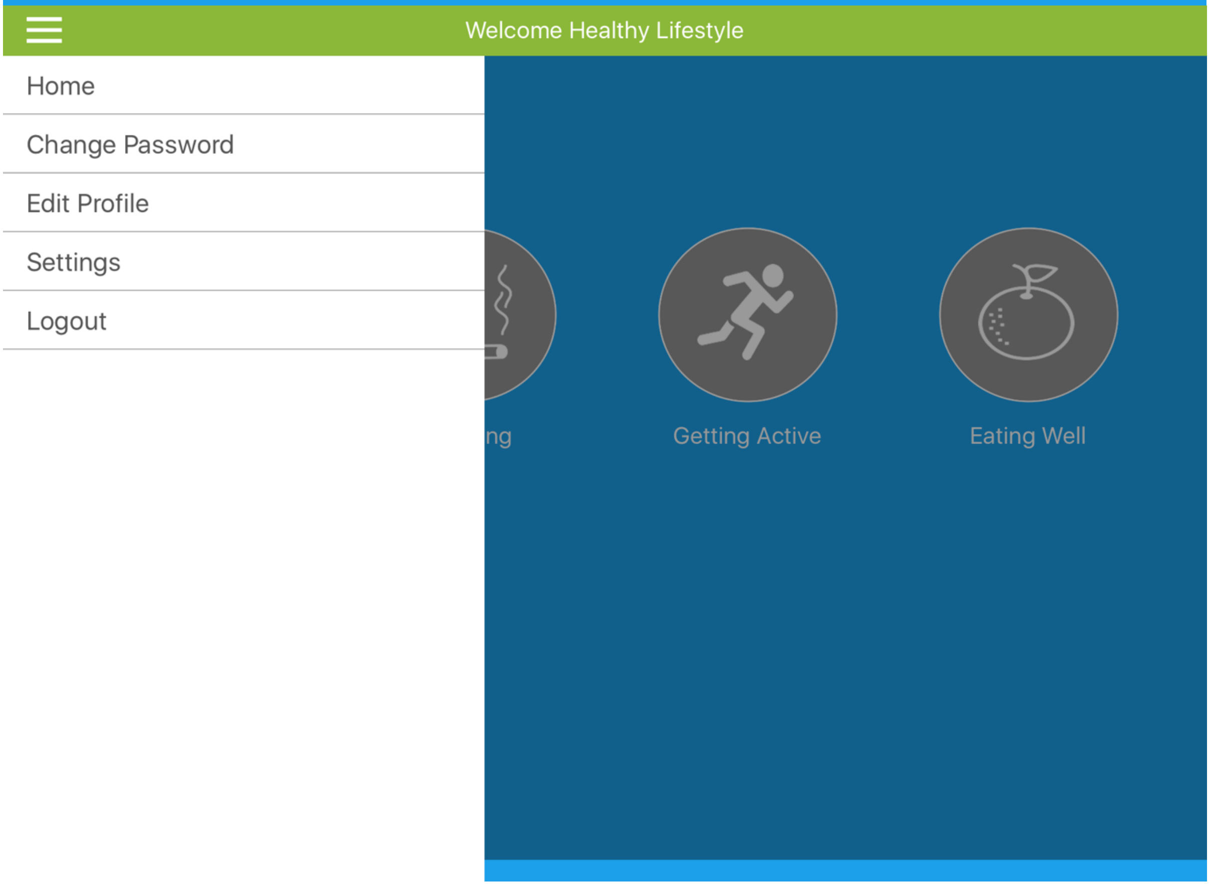
Welcome Healthy Lifestyle
Home
Change Password
Edit Profile
Settings
Logout
Getting Active
Eating Well

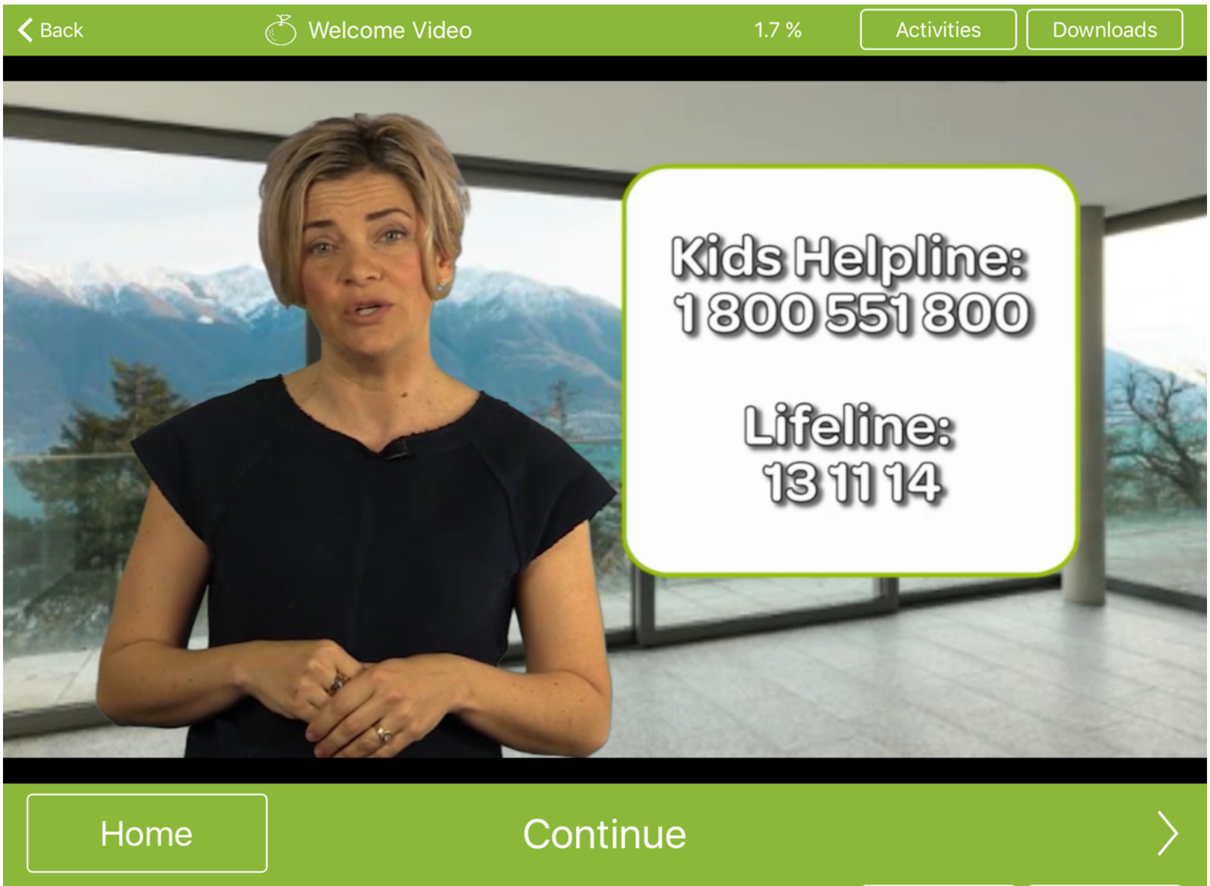
Back
Welcome Video
1.7 %
Activities
Downloads
Kids Helpline:
1 800 551 800
Lifeline:
13 11 14
Home
Continue

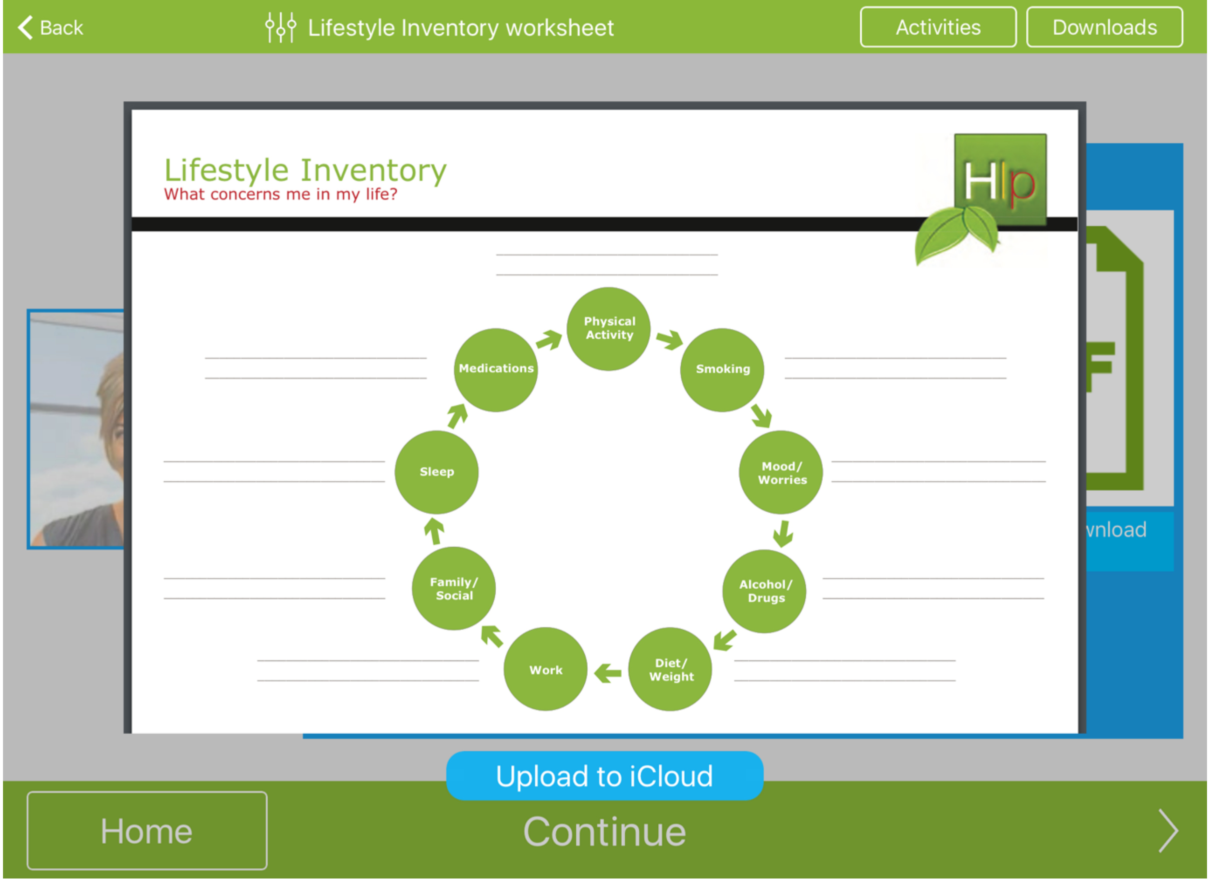
Back
Lifestyle Inventory worksheet
Activities
Downloads
Lifestyle Inventory
What concerns me in my life?
Physical Activity
Smoking
Mood/ Worries
Alcohol/ Drugs
Diet/ Weight
Work
Family/ Social
Sleep
Medications
Upload to iCloud
Home
Continue

Back Lifestyle Inventory worksheet Activities Downloads

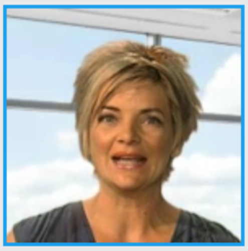

## Lifestyle Inventory

To help you with this process Please print out the following sheet and Let's have a chat about it all.

PDF

Click here to download this worksheet

Home Continue

Back Smoking 2 % Activities Downloads

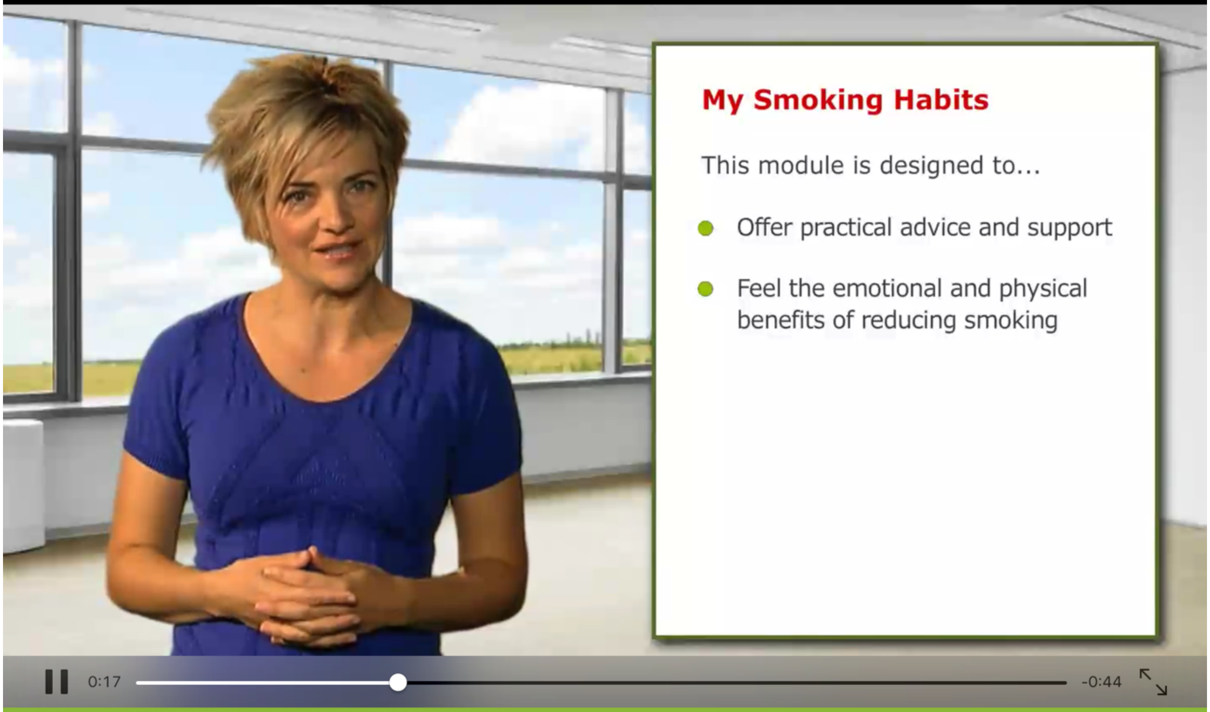


Home Continue

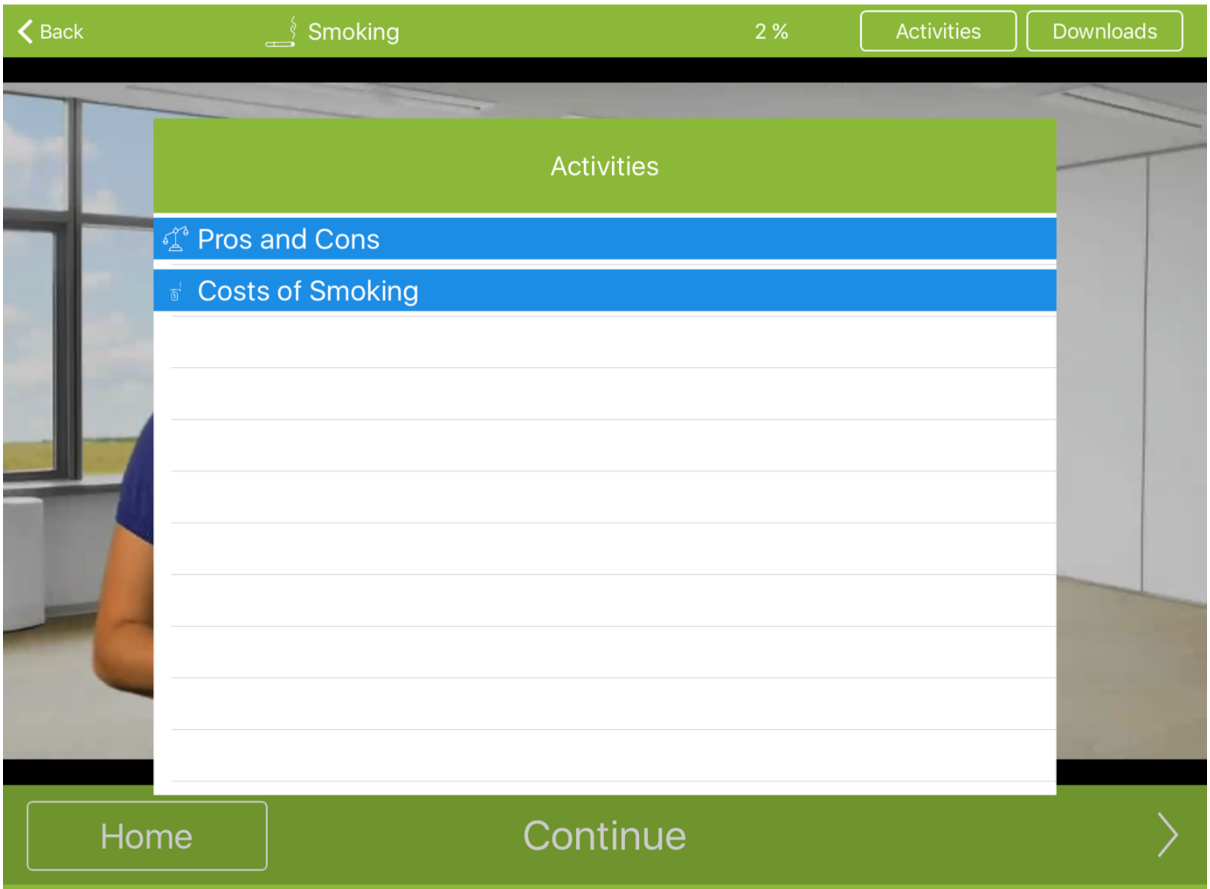
Back
Smoking
2 %
Activities
Downloads
Activities
Pros and Cons
Costs of Smoking
Home
Continue

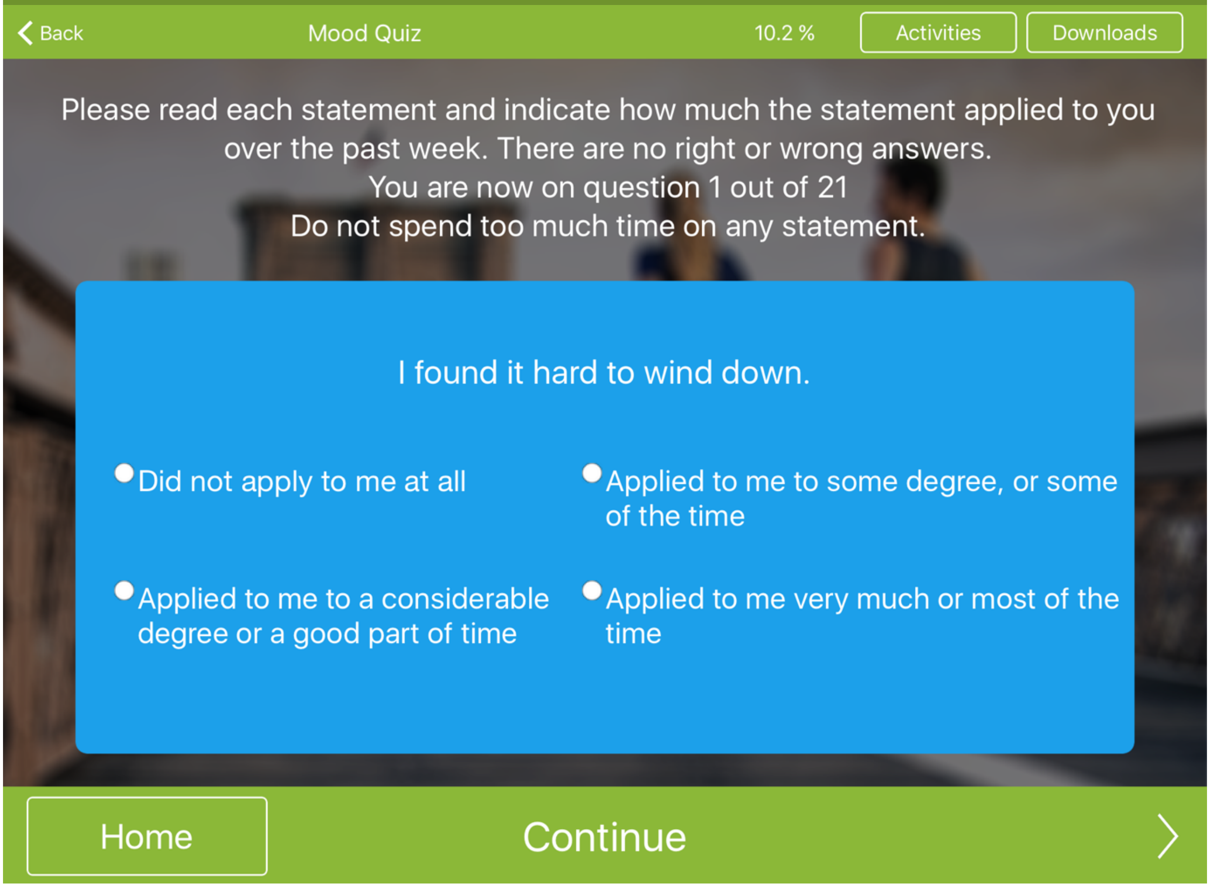
Back
Mood Quiz
10.2 %
Activities
Downloads
Please read each statement and indicate how much the statement applied to you over the past week. There are no right or wrong answers.
You are now on question 1 out of 21
Do not spend too much time on any statement.
I found it hard to wind down.
Did not apply to me at all
Applied to me to some degree, or some of the time
Applied to me to a considerable degree or a good part of time
Applied to me very much or most of the time
Home
Continue

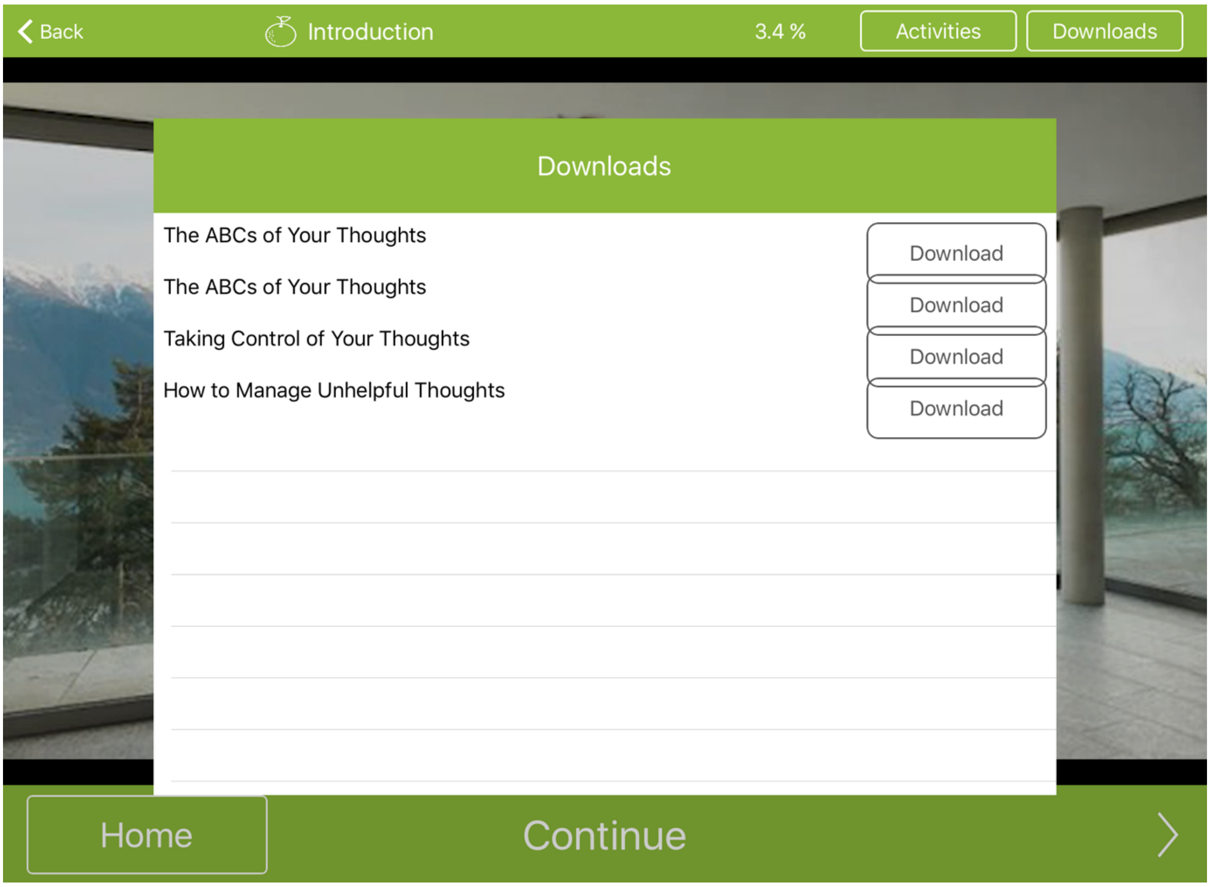
Back
Introduction
3.4 %
Activities
Downloads
Downloads
The ABCs of Your Thoughts
Download
The ABCs of Your Thoughts
Download
Taking Control of Your Thoughts
Download
How to Manage Unhelpful Thoughts
Download
Home
Continue
